# How a Clinician-Designed Digital Tool Improves Adherence to Measurement-Based Care and Clinical Outcomes in Psychiatry

**DOI:** 10.1192/j.eurpsy.2026.11339

**Published:** 2026-07-31

**Authors:** Y. Park, A. Vian-Lains, R. Frontini, C. Costa, S. Baptista, L. Nedel, F. Silva, A. Shahdid, N. Kennedy

**Affiliations:** 1 School of Medicine, RCSI; 2 Highfield Healthcare, Dublin, Ireland; 3 Neuroscience and Mental Health Clinic; 4 Internal Medicine, Red Cross Hospital, Lisbon, Portugal; 5 Ireland’s Centre for Applied AI, University College Dublin, Dublin, Ireland

## Abstract

**Introduction:**

Measurement-based care (MBC) uses patient-reported outcomes to guide treatment. Despite its robust evidence in improving engagement and outcomes it remains under-implemented due to time and workflow barriers. Tailored digital tools may enhance feasibility by automation of assessments and integration into workflows.

**Objectives:**

To describe the design, implementation and clinical outcomes of Psymap, a clinician-developed software supporting MBC implementation.

**Methods:**

Psymap is a web-based application that evolved from a Python script originally developed and refined over two years by one of the team’s psychiatrists. It streamlines MBC by integrating sociodemographic data, patient-reported outcome measures, and clinician-administered assessments into a single digital platform with automated scoring algorithms and interpretive text to support clinical evaluation. Psymap was introduced over 15 months in a private outpatient service. Patients completed validated digitized assessments. The tool produced patient-friendly summaries (Figure 1), automated documentation, and stored data securely. Baseline-to-endpoint (12 months or last observation) changes in PHQ9 and GAD7 were analysed. Patients who completed Psymap assessments at ≥90% of their visits were classified as High-Frequency Assessment (HFA) and the remainder as Low-Frequency Assessment (LFA). Kaplan–Meier and one-way ANOVA analyses compared rates of response (≥50% PHQ9 reduction) and remission (PHQ9<5), time to remission and mean score change across groups.

**Results:**

728 patients completed 1,798 assessments; 349 with ≥2 assessments were analysed. Psymap use at first visit rose from 37.7% to 96%. Higher use correlated with greater reductions in PHQ9 and GAD7 scores (r=–0.16, r=–0.18; p<.001). The HFA group (n=153) showed higher response (48% vs 33%) and remission (33% vs 19%) rates (χ^2^=8.9, χ^2^=8.1; p<.01). Kaplan-Meier analysis showed response and remission curves differed significantly (log-rank=5.9 and 6.0; p<.05); mean time to response was 80 vs 140 days. (Figure 2) Mean time to remission was 210 days for the HFA group, while the LFA group did not achieve 50% response. One-way ANOVA showed greater PHQ9 (–5.67 vs –4.08; F=4.55, p<.05) and GAD7 (–6.01 vs –3.89; F=9.16, p<.01) reductions in HFA vs LFA. (Figure 3) No adverse events were reported. Feedback highlighted improved clinician workflow and patient engagement.

**Image 1:**
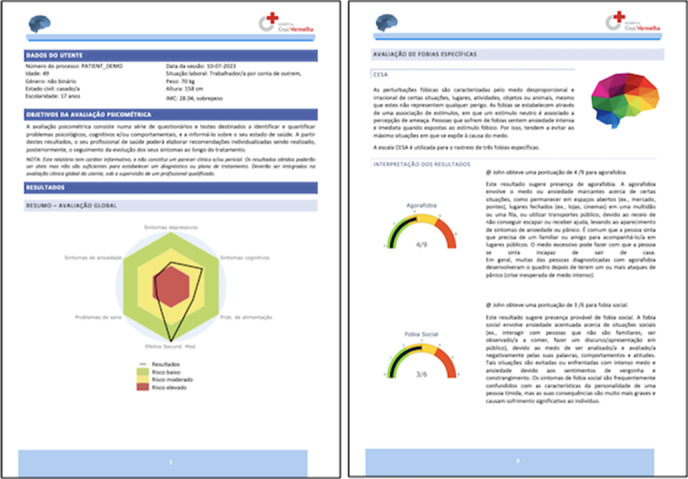
Image 1: Long description.

**Image 2:**
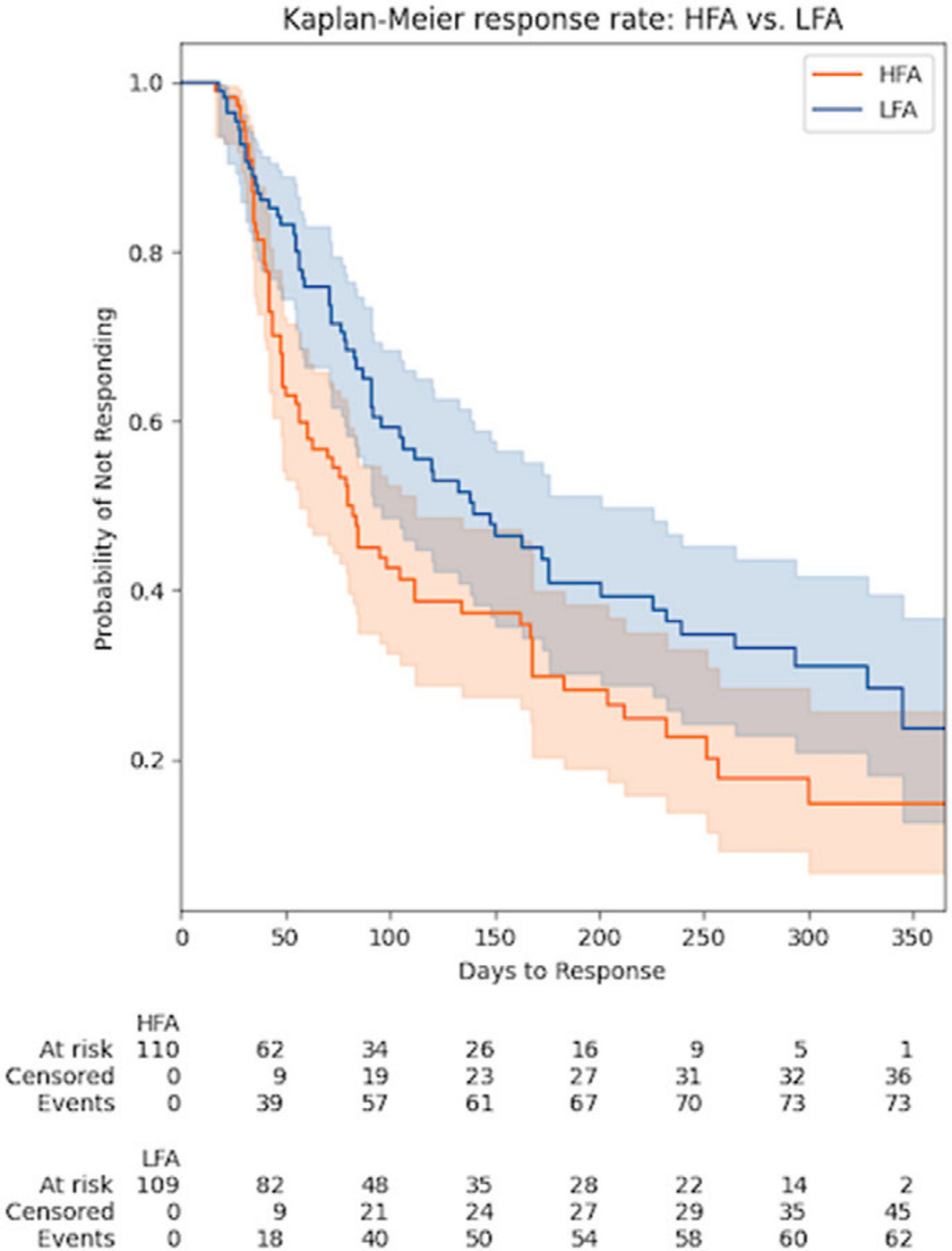
Image 2: Long description.

**Image 3:**
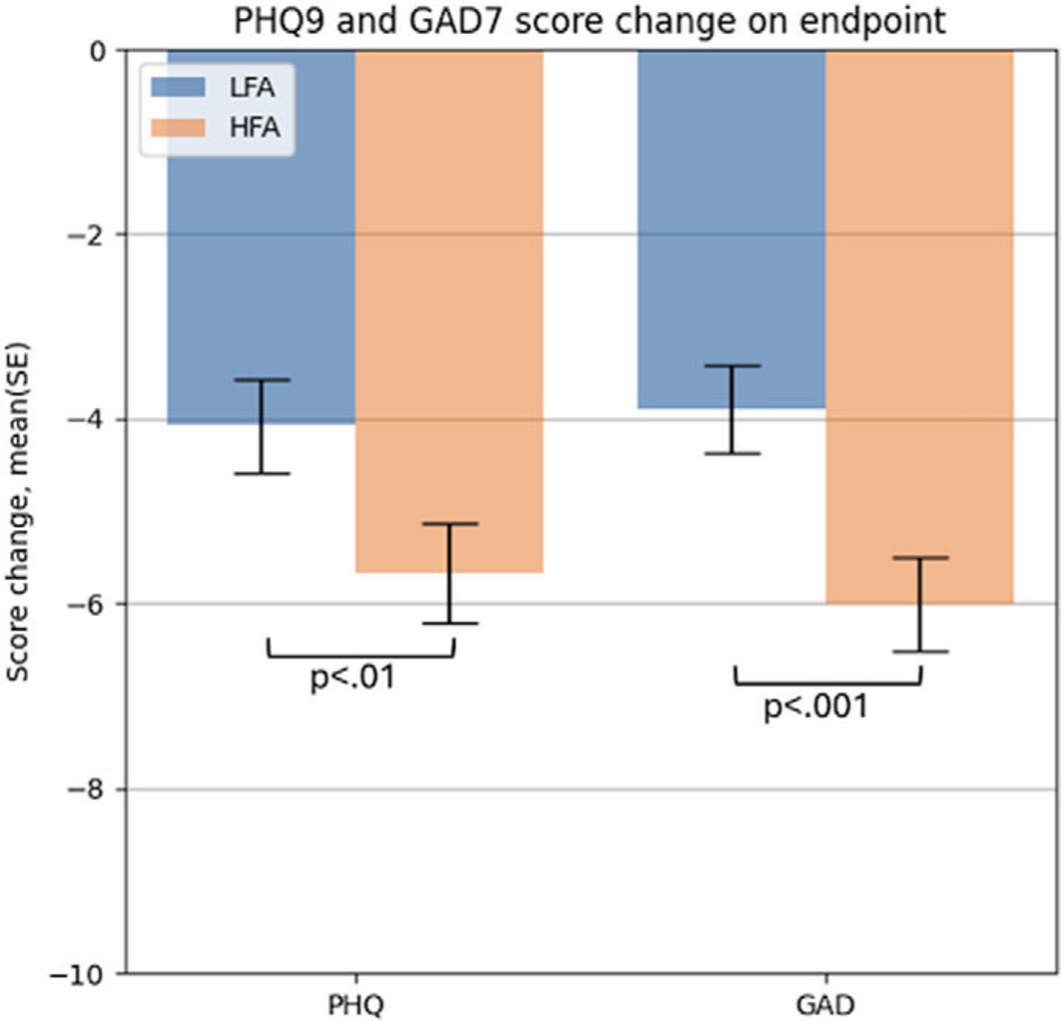
Image 3: Long description.

**Conclusions:**

Psymap is a feasible, scalable digital platform integrating MBC into routine practice. Consistent use was associated with improvement in depression and anxiety outcomes. Clinician-led, workflow-oriented digital tools may help overcome barriers to MBC implementation in psychiatry.

**Disclosure of Interest:**

Y. Park: None Declared, A. Vian-Lains Shareolder of: AVL developed and owns the Psymap software, R. Frontini: None Declared, C. Costa: None Declared, S. Baptista: None Declared, L. Nedel: None Declared, F. Silva: None Declared, A. Shahdid: None Declared, N. Kennedy: None Declared

## Long descriptions

### Image 1: Long description

A two-page document from Hospital Cruz Vermelha.

Page 1 contains patient data for P A T I E N T underscore D E M O and a section titled RESUMO - AVALIAÇÃO GLOBAL. This section features a hexagonal radar chart with six axes: Sintomas depressivos, Sintomas cognitivos, Prob. de alimentação, Efeitos Secund. Med., Problemas de sono, and Sintomas de ansiedade. The chart uses concentric color-coded zones: a red center for Risco elevado, a yellow middle ring for Risco moderado, and a green outer ring for Risco baixo. A black line plots the results, showing a peak in Sintomas depressivos and Sintomas de ansiedade.

Page 2 is titled AVALIAÇÃO DE FOBIAS ESPECÍFICAS and includes a C E S A section with a colorful brain icon. Below, under INTERPRETAÇÃO DOS RESULTADOS, are two semi-circular gauge charts.

1. Agorafobia: The gauge shows a score of 4/9, with the needle pointing to the yellow moderate risk zone.

2. Fobia Social: The gauge shows a score of 3/6, with the needle pointing to the yellow moderate risk zone.

Each gauge is accompanied by a paragraph of Portuguese text explaining the clinical implications of the scores.

Navigate back to Image 1:.

### Image 2: Long description

A Kaplan-Meier survival plot titled Kaplan-Meier response rate H F A vs. L F A. The y-axis is labeled Probability of Not Responding ranging from 0.0 to 1.0. The x-axis is labeled Days to Response ranging from 0 to 350.

Two stepped lines represent the data:

* An orange line for H F A shows a rapid decline in the probability of not responding, dropping below 0.4 by day 100 and ending near 0.15 at day 350. It is surrounded by a light orange shaded confidence interval.

* A blue line for L F A shows a slower decline, remaining above 0.6 at day 100 and ending near 0.25 at day 350. It is surrounded by a light blue shaded confidence interval.

Below the graph is a risk table with two sections:

* H F A group: At risk starts at 110 (day 0) and drops to 1 (day 350). Censored increases from 0 to 36. Events increase from 0 to 73.

* L F A group: At risk starts at 109 (day 0) and drops to 2 (day 350). Censored increases from 0 to 45. Events increase from 0 to 62.

Navigate back to Image 2:.

### Image 3: Long description

A bar chart titled P H Q 9 and G A D 7 score change on endpoint.

Axes and Legend:

* The y-axis is labeled Score change, mean S E and ranges from 0 at the top to negative 10 at the bottom.

* The x-axis has two categories: P H Q and G A D.

* The legend at the top left identifies two groups: L F A represented by blue bars and H F A represented by orange bars.

Data Trends:

* P H Q category: The L F A blue bar shows a mean change of approximately negative 4. The H F A orange bar shows a larger mean change of approximately negative 5.7. A bracket below these bars indicates a statistically significant difference with p less than .01.

* G A D category: The L F A blue bar shows a mean change of approximately negative 3.9. The H F A orange bar shows a larger mean change of approximately negative 6. A bracket below these bars indicates a statistically significant difference with p less than .001.

Error bars representing S E are present on all four bars, extending above and below the mean values.

Navigate back to Image 3:.

